# Impact of Immune-Inflammatory Microenvironment Alterations on the Bronchial Lumen of Children With Protracted Bacterial Bronchitis

**DOI:** 10.7759/cureus.20554

**Published:** 2021-12-20

**Authors:** Despoina Ntesou, Konstantinos Douros, Evangelos Tsiambas, Sotirios Maipas, Helen Sarlanis, Andreas C Lazaris, Nikolaos Kavantzas

**Affiliations:** 1 Department of Laboratory Medicine, University of West Attica, Egaleo, GRC; 2 Third Department of Pediatrics, National and Kapodistrian University of Athens School of Medicine, Athens, GRC; 3 Department of Cytopathology, 417 Army Equity Fund Hospital (NIMTS), Athens, GRC; 4 Pathology, National and Kapodistrian University of Athens School of Medicine, Athens, GRC; 5 Pathology, National and Kapodistrian University of Athens, Athens, GRC

**Keywords:** basement membrane thickening, chronic cough, immunology, asthma, protracted bacterial bronchitis

## Abstract

Protracted bacterial bronchitis is a syndrome that is among the most common causes of chronic cough. In order to understand its pathogenetic mechanisms, there is an increasing interest in the study of the immune microenvironment in the bronchial lumen. The aim of this retrospective study is the determination of the types and quantity of the inflammatory cells that infiltrate the bronchial epithelium as well as of the thickness of the basement membrane. Ninety-seven endobronchial biopsies, obtained from 77 children (30 males and 47 females) aged between 5 and 14 years, with chronic (>8 weeks) wet/productive cough, were subjected to hematoxylin and eosin staining. Using an appropriate image analysis and processing software, we determined the types and the quantity of the inflammatory cells that infiltrated the bronchial epithelium, and the thickness of the basement membrane. The metric data were then subjected to extensive statistical analysis. According to our results, females had increased levels of eosinophils (p = 0.021) and lymphocytes (p = 0.044) compared to males. Moreover, we found that membrane thickness was negatively correlated with the number of eosinophils (p < 0.0001), neutrophils (p = 0.023), and lymphocytes (p = 0.024). Finally, the pairwise comparisons of the number of eosinophils, neutrophils, lymphocytes, and other cell types revealed significant (p < 0.05) positive correlations. Protracted bacterial bronchitis activates pulmonary innate immune pathways. Also, it is accompanied by basement membrane thickening, which is a typical characteristic of several respiratory diseases, such as asthma.

## Introduction

Chronic cough in children was for many years a challenging situation in terms of understanding and management. Misdiagnoses and subsequent inappropriate therapeutic strategies were common phenomena, since cough was initially attributed to bronchitis, and treated with antibiotics, and later it was wrongly linked to asthma resulting in an explosion of asthma overdiagnosis and overtreatment [1]. It was only in 2006 when a study conducted in Australia introduced the term “Protracted Bacterial Bronchitis” (PBB) to describe a syndrome that is among the most common causes of chronic cough [2]. Although PBB-like conditions and the corresponding clinical features have been well described during the last decades, the aforementioned study paved the way for the recognition of PBB as a distinct diagnostic entity that needs to be studied in order to decipher its clinical and pathobiological features.

The initial definition of PBB included three criteria: a) A history of chronic wet/productive cough, b) positive bronchoalveolar (BAL) cultures for a respiratory pathogen, and c) response to a 2-week course of antibiotics [2]. Nowadays, PBB is an umbrella term that includes four categories: a) PPB-micro represents the initial definition, b) PBB-clinical includes the presence of chronic wet cough, absence of symptoms of other conditions that are related with wet/productive cough and symptoms resolution after a 2-week course of oral antibiotic, c) PBB-extended includes the aforementioned categories but resolution needs a 4-week administration of antibiotics, and d) recurrent PBB is the occurrence of more than three PBB-micro or -clinical events per year [3,4].

From a clinical point of view, PBB is related with high morbidity and decreased quality of life, and, if untreated, PBB can lead to chronic suppurative endobronchial lung disease, and eventually to bronchiectasis [5]. PBB is mainly manifested with wet cough, which, in general, is the most prominent first thing in the morning, and it may or may not be accompanied by shortness of breath during exercise, tiredness, lack of energy, and wheezing. However, children suffering from PBB look healthy without growth and developmental abnormalities [1]. From a pathobiological point of view, PBB is associated with aberrant mucociliary clearance, and with the formation of bacterial biofilm in the lower airways [6,7].This biofilm enhances survival of bacteria in this area, since it acts as a matrix which facilitates the adherence and the access to nutrients, preventing in parallel antibiotic penetration [1]. Hereto, the most common bacteria found in children with PBB is non-typeable *Haemophilus influenzae* (38-81%), followed by *Streptococcus pneumoniae* (16-39%) and *Moraxella catarrhalis* (19-51%) [3]. Finally, Wurzel et al. (2014) examined the presence of viruses in BAL samples from children with PBB and found that the most common virus was human adenovirus [8].

By the virtue that nowadays there is an increasing interest in the study of the immune microenvironment in the bronchial lumen in order to understand the pathogenetic mechanisms of PBB, we aimed at determining the types and the quantity of the inflammatory cells that infiltrate the bronchial epithelium, and the thickness of the basement membrane.

## Materials and methods

The study cohort consisted of 97 bioptic sections of bronchial mucosa that were processed at the First Department of Pathology of the Medical School of the National and Kapodistrian University of Athens. The material of the endobronchial biopsy was obtained from 77 children (30 males and 47 females) with chronic (>8 weeks) wet/productive cough. The children’s age ranged between 5 and 14 years. The research procedures of this retrospective study comply with the ethical standards of the World Medical Association Declaration of Helsinki. Approval for the use of the bioptic material was acquired from the Bioethics and Deontology Committee of the National and Kapodistrian University of Athens (Protocol number of the approval: 1617006414). Informed consent was obtained from all study participants.

Each paraffin-embedded section (depth: 4 μm) was subjected to hematoxylin and eosin (H&E) staining. H&E staining is the most popular and one of the principal stains in histology. It provides an overview of the tissue and its structure. Hematoxylin, which has a deep blue-purple color, is a basic dye and binds with basophilic (negatively charged) nuclear components like DNA and RNA. In that way the nucleus, where these macromolecules are located, stains up in one color (purple). On the other hand, eosin is a fluorescent red dye, which is negatively charged. As an acidic dye, eosin stains basic structures of a cell giving them a red or a pink color. Therefore, cytoplasm, which is positively charged, uptakes the eosin dye and it is stained pink [9].

The procedure consisted of four steps: a) Deparaffinize and rehydration, b) hematoxylin staining, c) eosin staining and dehydration, and d) clearing and cover-slipping. In more detail, during the first step, each slide was placed sequentially in xylene, 100%, 95%, and 80% ethanol, and finally in deionized water. Afterwards, for the hematoxylin staining the slide was initially placed in hematoxylin for 3 min and it was rinsed with deionized water, then dipped in acid ethanol, and finally rinsed with water. Then the slide was placed sequentially in eosin for 30 s, in 95% and 100% ethanol, and finally in xylene. The procedure was completed with the slide cover-slipping.

In the next step of the study, we observed the slides under microscope (Olympus BX51), and we analyzed the corresponding pictures using an appropriate image analysis and processing software (Media Cybernetics Image Pro Plus), in order to determine the types and the quantity of the inflammatory cells that infiltrate the bronchial epithelium, and the thickness of the basement membrane. For the first aim, we determined the morphological parameters of the cells, such as area, diameter, perimeter, and optical density, per area equaling to 1 mm^2^. The thickness of the basement membrane was assessed by drawing the two opposite perimetrical lines and by automatically estimating the distance between them.

The metric data were subjected to biostatistical analysis using the SPSS software program (IBM Corp. Released 2011. IBM SPSS Statistics for Windows, Version 20.0. Armonk, NY: IBM Corp). The correlation of these data with patients’ gender was performed using the Student’s t-test. Moreover, the correlations between continuous variables of the study were examined with Pearson’s correlation coefficient (r). A p-value of less than 0.05 was considered as an indication of statistical significance.

## Results

Microscopic observation

**Figure 1:**
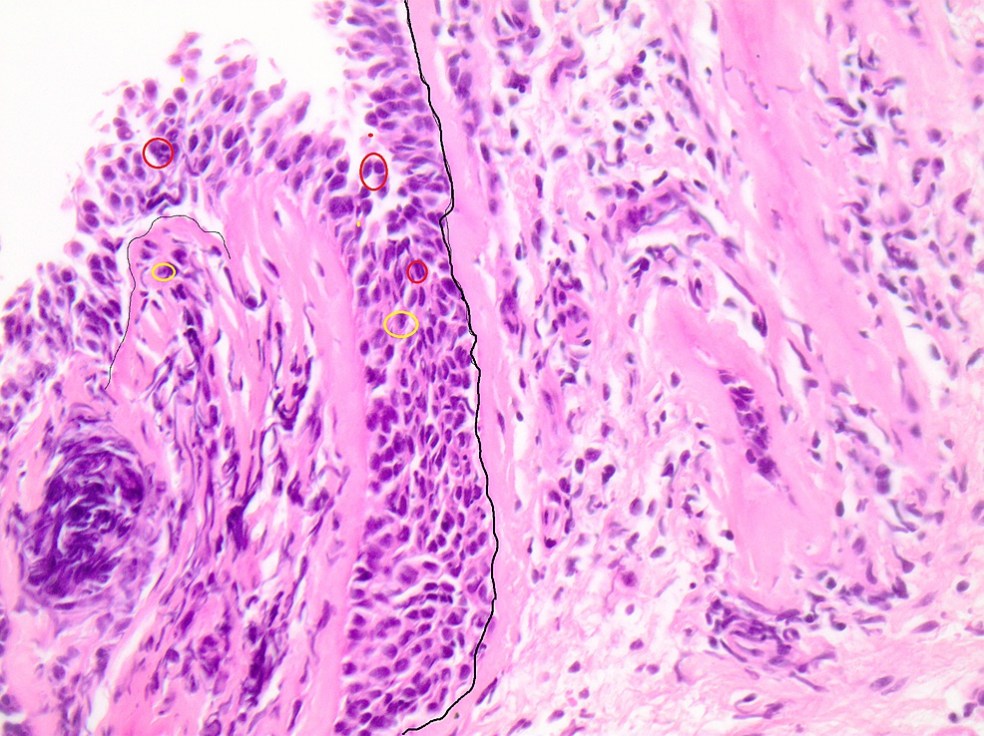
Inflammatory infiltration of bronchial epithelia in a PBB tissue section A variety of mixed inflammatory cell populations penetrate the epithelium substrate. Recruitment of eosinophils, neutrophils, and lymphocytes in PBB tissue is a significant histopathological feature. Especially, intense neutrophilic inflammation is predominant (neutrophils in yellow circles, lymphocytes and eosinophils in red circles, original magnification: 100x). PBB, protracted bacterial bronchitis.

**Figure 2:**
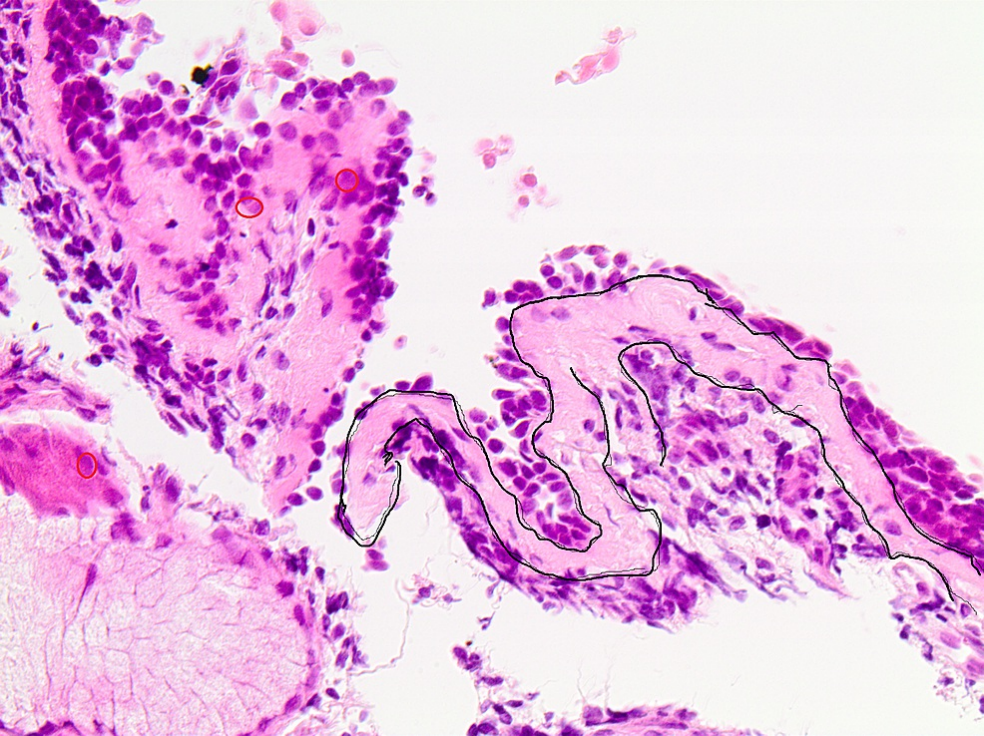
Representative picture that shows the thickness of the basement membrane Note the increased pale purple area inside the continuous black line. Basement membrane thickening is among the typical features of asthmatic airways and it is considered as the main reason for the observed airway hyper-responsiveness which results in airflow obstruction (original magnification: 100x).

Following the aforementioned procedure, we took representative pictures of appropriate areas in order to determine the inflammatory cells that infiltrate the bronchial epithelium (Figure 1) as well as the thickness of the basement membrane (Figure 2).

Association of patients’ gender with cell number and membrane thickness

According to Student’s t-test, patients’ gender was significantly associated with the number of eosinophils and lymphocytes, since it was observed that females had increased levels of these cell types compared to males. In more detail, regarding the eosinophils, the mean value in female children was 11.85, whereas the corresponding value in male patients was 8.63 (p = 0.021, 95% CI = -5.94 to -0.50). Similarly, the mean value of lymphocytes in females and males was 13.96 and 11.37, respectively (p = 0.044, 95% CI = -5.12 to -0.07). On the contrary, no significant association was found between patients’ gender and the membrane thickness as well as the number of other inflammatory cells (Table 1).

**Table 1:** Association of patients’ gender with cell number and membrane thickness ^a^SD: standard deviation; ^b^95% CI: 95% confidence interval of the difference. *Calculated by the Student’s t-test.

	Gender	N	Mean ± SD^a^	95% CI^b^	p-Value*
Eosinophils	Male	30	8.63 ± 5.04	-5.94 to -0.50	0.021
	Female	47	11.85 ± 6.29		
Neutrophils	Male	30	8.97 ± 4.96	-2.97 to 1.20	0.40
	Female	47	9.85 ± 4.16		
Lymphocytes	Male	30	11.37 ± 6.02	-5.12 to -0.07	0.044
	Female	47	13.96 ± 5.01		
Others	Male	30	15.7 ± 8.01	-6.93 to 1.05	0.14
	Female	47	18.6 ± 8.90		
Thickness	Male	30	5.27 ± 2.61	-0.08 to 1.17	0.70
	Female	47	5.08 ± 1.74		

Correlation of patients’ age with cell number and membrane thickness

**Figure 3:**
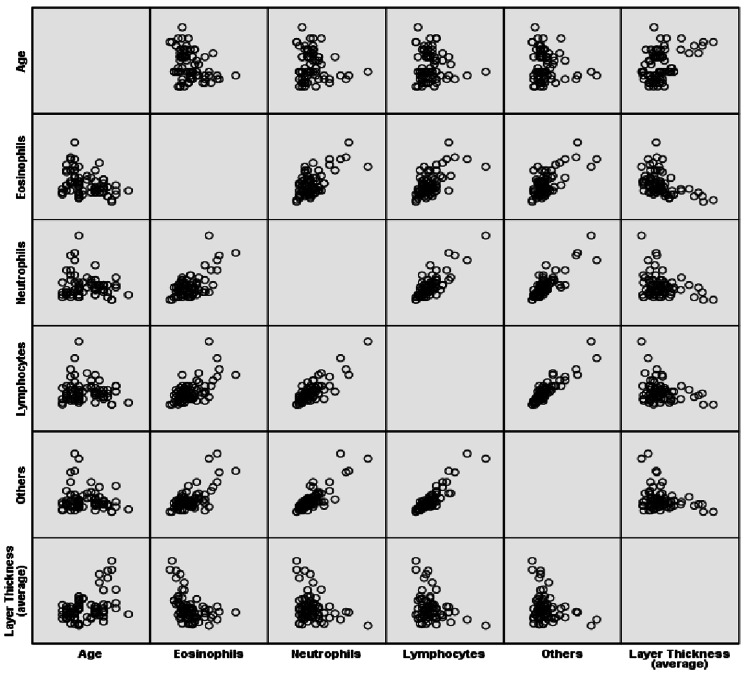
Correlation of the number of the studied inflammatory cells and the thickness of basement membrane with age The pairwise comparisons of the number of eosinophils, neutrophils, lymphocytes, and other cell types revealed significant positive correlations. Membrane thickness was negatively correlated with the number of eosinophils, neutrophils, and lymphocytes although it was positively correlated with the patients’ age.

By performing Pearson’s correlation coefficient, we found that the membrane thickness was negatively correlated with the number of eosinophils (p < 0.0001, r = -0.448), neutrophils (p = 0.023, r = -0.260), and lymphocytes (p = 0.024, r = -0,256). However, it was positively correlated with the patients’ age (p < 0.0001, r = 0.433). Moreover, a significant negative correlation of age with the number of eosinophils (p = 0.002, r = -0.340) was observed. The pairwise comparisons of the number of eosinophils, neutrophils, lymphocytes, and other cell types revealed significant positive correlations. More specifically, a number of eosinophil cells were positively correlated with neutrophils (p < 0.0001, r = 0.625), lymphocyte cells (p < 0.0001, r = 0.585), and other cells (p < 0.0001, r = 0.669). Accordingly, the number of neutrophil cells demonstrated a significant positive correlation with lymphocytes (p < 0.0001, r = 0.821) and other cells (p < 0.0001, r = 0.828). Finally, lymphocyte cells were also positively correlated with other cells (p < 0.0001, r = 0.903) (Figure 3).

## Discussion

According to several studies, PBB seems to be the most frequent cause of chronic wet cough in pre-school children. More in particular, it has been reported that the disease prevalence in children with chronic cough is about 40% [10,11]. However, many believe that this percentage is underestimated due to the limited awareness of the condition, which frequently leads to misdiagnosis as asthma. In fact, according to Craven and Everard (2013), almost 55% of children with persistent cough were initially diagnosed with asthma, but after a thorough investigation, the diagnosis changed to PBB in 40% of cases [10]. Recently, a study group reported high levels of PBB in children referred to tertiary respiratory care with chronic cough [12].

Nowadays, there is scarce knowledge regarding the pathophysiological mechanisms of PBB. More specifically, the most prevalent theory is a the vicious circle hypothesis according to which respiratory infections caused by bacteria such as *Haemophilus influenzae*, *Moraxella catarrhalis*, and *Streptococcus pneumoniae* result in a malfunction of airway epithelial cilia and decreased removal of secretions, leading thereby to chronic bacterial infection. The bacteria then form a biofilm that enhances the bacterial survival and proliferation, deteriorating, thus, the bacterial infection [12,13]. As a result, bacteria can survive on the airway mucosa and cause chronic bronchitis that is accompanied by intense neutrophilic inflammation [11,14]. The activation of inflammatory reaction has triggered the research regarding the immune microenvironment of PBB-affected children in order to clarify the pathophysiology of this condition.

Taking into consideration the above-mentioned data along with the several lines of evidence reporting that basement membrane thickness is one of the pathological characteristics of asthma and other chronic respiratory diseases [15-17], we determined the types and the quantity of the inflammatory cells that infiltrate the bronchial epithelium, and the thickness of the basement membrane.

The microscopic observation of the H&E-stained slides obtained from children with PBB revealed the recruitment of eosinophils, neutrophils, and lymphocytes. The presence of neutrophils is in accordance with the observation that the lower airway profile of PBB is characterized by intense neutrophilic inflammation. Moreover, elevated expression levels of toll-like receptors (TLRs) TLR2 and TLR4, human b-defensin-2, and mannose-binding lectin in BAL specimens obtained from children with PBB have been reported. These findings reinforce the idea of the activation of epithelial-/neutrophil-mediated pathogen recognition and clearance mechanisms in PBB [2,11,18,19]. Moreover, the statistical analysis revealed significant positive correlations among the number of eosinophils, neutrophils, and lymphocytes. In more detail, the number of eosinophil cells was positively correlated with neutrophil (p < 0.0001, r = 0.625) and lymphocyte cells (p < 0.0001, r = 0.585), whereas the number of neutrophil cells demonstrated a significant positive correlation with lymphocyte cells (p < 0.0001, r = 0.821). These results are also indicative of the activation of pulmonary innate immune pathways.

Regarding the thickness of the basement membrane, the microscopic observation revealed thickening of the membrane, which is indicative of the remodeling of the airway wall structure. Basement membrane thickening is among the typical features of asthmatic airways and it is considered as the main reason for the observed airway hyper-responsiveness which results in airflow obstruction [16,20]. The fact that asthma and PBB share several common symptoms and the fact that PBB is often misdiagnosed as asthma can justify the fact that children with PBB are characterized by basement membrane thickening. Interestingly, the impact of chronic irritation of the bronchial epithelia due to bronchiectasis, recurrent PBB, and asthma in children combined or not with PBB is under investigation. A study group analyzed a series of children by monitoring them for a significant period of time and they concluded that they should be carefully followed up clinically since a non-negligible percentage of them may end up with bronchiectasis [21,22]. Additionally, another study group focused on the obstructive sleep apnea (OSA) evaluation by implementing polysomnography in children as a reliable parameter for the severity of the corresponding bronchial-dependent diseases. They observed that asthma and PBB - as the most frequent etio-pathogenetic factors - demonstrate significant altered levels of OSA [23].

Undoubtedly, the current study has limitations. For example, it would be interesting to include the healthy controls in order to determine the variations in the number of immune cells and the thickness of the basement membrane between children with PBB and the healthy ones. It would also be informative to carry out a comparative study of patients with PBB and asthma in order to identify factors that can aim to the differential diagnosis of these respiratory diseases. Finally, we deem mandatory the external validation of our results on larger, independent, and multicentric cohorts in order to verify our observations.

## Conclusions

PBB is a main and frequent cause of chronic wet cough in pre-school children. It is also well known that bronchial epithelia chronic irritation due to bronchiectasis, recurrent PBB, and asthma in children - combined or not with PBB - is a crucial parameter for the severity of the corresponding histopathological lesions. According to our results, PBB activates pulmonary innate immune pathways. There are significant positive correlations among the number of eosinophils, neutrophils, and lymphocytes. The lower airway profile of PBB is characterized especially by intense neutrophilic inflammation. Based on their increased numbers in PBB specimens, there is a correlation with enhanced activation of different pulmonary innate immune pathways. Furthermore, basement membrane thickening is a critical issue for the normal function of asthmatic airways leading to a progressive airway hyper-responsiveness and airflow obstruction. We showed that PBB is accompanied by basement membrane thickening, which is a typical characteristic of several respiratory diseases, such as asthma. However, given the limitations of our study, further research is required to support our observations.
